# HIV viraemia during pregnancy in women receiving preconception antiretroviral therapy in KwaDukuza, KwaZulu-Natal

**DOI:** 10.4102/sajhivmed.v20i1.847

**Published:** 2019-04-10

**Authors:** Vuyokazi Ntlantsana, Richard J. Hift, Wendy P. Mphatswe

**Affiliations:** 1 Department of Obstetrics and Gynaecology, School of Clinical Medicine, University of KwaZulu-Natal, Durban, South Africa; 2 School of Clinical Medicine, University of KwaZulu-Natal, Durban, South Africa

## Abstract

**Background:**

Preconception antiretroviral therapy (PCART) followed by sustained viral suppression is effective in preventing mother-to-child transmission of HIV. The rates of persistent and transient viraemia in such patients have not been prospectively assessed in South Africa.

**Objectives:**

We determined the prevalence of transient and persistent viraemia in HIV-positive women entering antenatal care on PCART and studied variables associated with viraemia.

**Methods:**

We performed a prospective cross-sectional observational study of HIV-positive pregnant women presenting to a primary healthcare facility in KwaZulu-Natal. All had received at least 6 months of first-line PCART. Viral load (VL) was measured, patients were interviewed, adherence estimated using a visual analogue scale and adherence counselling provided. Viral load was repeated after 4 weeks where baseline VL exceeded 50 copies/mL.

**Results:**

We enrolled 82 participants. Of them, 59 (72%) pregnancies were unplanned. Fifteen participants (18.3%) were viraemic at presentation with VL > 50 copies/mL. Of these, seven (8.5%) had viral suppression (VL < 50 copies/mL), and eight remained viraemic at the second visit. Adherence correlated significantly with viraemia at baseline. Level of knowledge correlated with adherence but not with lack of viral suppression at baseline. Socio-economic indicators did not correlate with viraemia. No instances of vertical transmission were observed at birth.

**Conclusions:**

Approximately 20% of women receiving PCART may demonstrate viraemia. Half of these may be transient. Poor adherence is associated with viraemia, and efforts to encourage and monitor adherence are essential. The rate of unplanned pregnancies is high, and antiretroviral therapy programmes should focus on family planning needs of women in the reproductive age group to prevent viral non-suppression prior to pregnancy.

**Keywords:**

Preconception Antiretroviral Therapy; HIV; Viraemia; Antenatal Care; Adherence.

## Introduction

Approximately 35 million people in the world are infected with HIV. Sub-Saharan Africa accounts for 71% of these infections.^1^ Women account for 51% of the world’s HIV infections,^2^ and 90% of HIV infections in children below 15 years of age are a result of mother-to-child transmission (MTCT).^3^ The HIV prevalence among South African women attending antenatal clinics (ANCs) in 2013 was reported as 29.9% in South Africa and as 37.4% in the province of KwaZulu-Natal.^4^ Maternal viral load (VL) is one of the main determinants of MTCT.^5^ Studying the magnitude of the problem of viraemia in pregnancy and identifying factors associated with this viraemia is important if future generations are to be born HIV-free.

VL monitoring is the best means of predicting clinical outcomes and of measuring the effectiveness of antiretroviral therapy (ART) programmes.^6^ Patients who respond successfully to ART will normally demonstrate undetectable VLs. In some cases, virus may, however, be detectable during therapy. We define low-level viraemia (LLV) as detectible viraemia above 50 copies/mL and below 1000 copies/mL, as defined by Hermans et al.^7^ Low-level viraemia suggests ongoing HIV replication and is associated with increased risk of eventual virological failure.^7^ Drug resistance and non-adherence are important causes of persistent viraemia in patients receiving ART.^8^ Drug resistance and, therefore, persistent viraemia is associated with previous exposure to ART or to antiretroviral drugs given as part of prevention of MTCT (PMTCT) programmes, particularly where the patients were exposed to less-effective regimens, long-term exposure to ART and primary infection with resistant strains of virus.^9^

Transient viraemia is defined as an episode of LLV that occurs in the presence of effective ART and is confirmed by viral suppression before and after the period of viraemia, with measurements taken at least 30 days apart.^10,11^ Although transient viraemia has been attributed to immune activation of latently infected cells, resulting in release of HIV RNA particles with ongoing replication of both antiretroviral-sensitive and antiretroviral-resistant virus,^12^ recent studies suggest that immune activation may in fact be established before ART initiation and persist during viral suppression and is therefore not responsible for LLV.^13^ High pretreatment VL has been associated with higher incidence of transient viraemia as viral reservoirs tend to be higher.^11^ Transient viraemia is associated with increased risk of viral resistance and eventual virological failure, particularly if recurrent.^11,14,15^

The United Nations Programme on HIV/AIDS set a goal of 90% of viral suppression in patients receiving ART by 2020.^16,17^ Current South African performance falls short of this. Recent prospective studies of patients receiving ART have found LLV rates varying from 18% to 31%.^7,17,18^ Approximately half of these patients with LLV eventually develop virological treatment failure.^7^ When maternal viraemia is completely suppressed throughout pregnancy, the rate of MTCT is reduced to below 0.5%,^19^ whereas the rate of transmission in the absence of therapy lies between 15% and 45%.^7,20^

In the mother receiving ART, higher perinatal transmission rates occur with LLV than when the VL is below 50 copies/mL,^15^ and the risk is increased where episodes of viraemia occur during the pregnancy.^21^ Adequate VL suppression reduces the MTCT risk during vaginal delivery to a level where caesarean section offers no advantage.^22^

Data from low, middle and high-income countries show that by 2011, only about 72% of pregnant women achieved the desirable adherence of 80% to ARVs, with adherence deteriorating in the post-partum period.^8,23,24^ Though previous studies of viraemia in pregnant South African women receiving ART were cross-sectional, Myer et al. studied a group of pregnant HIV-positive women initiated on ART prospectively.^25^ They reported that 23% failed to achieve viral suppression during pregnancy. Of the 77% who were initially suppressed, 6% subsequently developed viraemia, half of whom proved transient whereas the other half developed virological treatment failure. The study noted a striking loss of suppression in the post-partum period. Only 70% of the original cohort that started on ART during pregnancy maintained consistent viral suppression up to 12 months post-partum.^26^

Reported rates of detectable VL at the time of delivery in patients on ART vary. Cragg et al. reported a rate of 3% in women who enter ANC already on ART,^27^ while Myer et al. reported a rate of 27% in patients who initiated ART during pregnancy.^25^ A South African study has shown a correlation between maternal VL at birth and transmission, with the risk being 0.25%, 2% and 8.5%, respectively, with VLs lower than 50, of 50–1000 and more than 1000 copies/mL.^25^

Preconception antiretroviral therapy (PCART) is more effective than ART commenced after conception in reducing MTCT, and it is associated with substantial decreases in MTCT.^27,28^ Recently, zero transmission rates have been reported from South Africa^29^ and Burkina Faso.^30^ Although Omole et al. reported a significantly higher rate of viraemia in pregnant South African women compared with non-pregnant women in the period 2007–2008,^31^ current opinion holds that pregnancy itself does not impact adversely on VL, whether via physiological or socio-behavioural mechanisms, including adherence.^23,32,33^

No single study has prospectively followed South African patients on PCART, differentiated transient from persistent viraemia and attempted to correlate these with factors such as adherence and socio-economic indicators. This study was therefore undertaken to quantify the rate of HIV viraemia in pregnant women on PCART, to determine the proportion of women with transient viraemia versus persistent viraemia, to determine factors associated with viraemia and to determine birth HIV status of children born to women on PCART.

## Methods

### Study design

We conducted a cross-sectional observational study where we studied a group of pregnant HIV-positive women who attended the ANC of a primary healthcare facility in the semirural KwaDukuza District of KwaZulu-Natal. Participants were enrolled between November 2016 and April 2017. Pregnant women aged 18 years of age or older who had received ART for a minimum of 24 weeks before conception and whose gestational age did not exceed 30 weeks were invited to participate. Patients on second-line ART were excluded.

On the assumption that the proportion of patients with viraemia at first presentation is 13%, as previously reported in Cape Town,^34^ we predicted that a sample size of 89 patients was necessary to determine the prevalence of viraemia in our participants with a 95% confidence level and an error margin of 7%.

### Data collection

A blood sample for baseline VL was drawn at the first antenatal booking visit. Data were collected by questionnaire, administered by the primary researcher (VN), who is fluent in Zulu, which facilitated the process as all participants were first-language Zulu speakers. Questions were designed to cover past ART history, past PMTCT treatment exposure, sexual history, partner’s HIV status and exposure to ART, reproductive health history and a range of socio-economic indicators. The questionnaire was piloted by the researcher. A structured self-adherence questionnaire adapted from the AIDS Counselling and Treatment Group baseline adherence questionnaire^35^ with a visual analogue scale (VAS) was administered as a measure of adherence. All participants were then counselled on adherence and instructed to bring their pills at the next visit for a pill count.

Patients returned one week after the initial interview. A pill count was performed and they were informed of their initial VL results. Participants with any detectable VL received additional adherence counselling.

All participants were requested to return for a final visit after four weeks to repeat the pill count and perform a follow-up VAS. A second VL sample was drawn on those who had a baseline viraemia above 50 copies/mL. Persistently viraemic participants with a VL exceeding 1000 copies/mL were switched to second-line ART according to standard treatment protocols. For the purposes of this study, viral suppression was defined as a VL less than 50 copies/mL, transient viraemia was defined as a VL at any level above 50 copies/mL on the initial test but where viral suppression was achieved on the repeat test, and persistent viraemia as a VL above 50 copies/mL, present on both the initial and the repeat test.

Blood was drawn into EDTA-containing tubes and transported to the National Health Laboratory Service (NHLS), where HIV VL testing was conducted by the Cobas^®^ 8800/6800 HIV-1 test methodology. In the case of neonates, whole blood was collected at birth onto filter cards and then air dried as dried blood spots. Samples were stored on-site in a refrigerator and transported to the regional reference laboratory within the time frame recommend by the NHLS. HIV polymerase chain reaction (PCR) testing was performed using the Roche COBAS^®^ TaqMan^®^ HIV-1 Qualitative Test Version 2 (Roche Molecular Systems, Inc., Branchburg, NJ, USA).

Pregnancy outcomes were determined by accessing the clinic records to review the postnatal consultation. Where necessary, patient records were also accessed from the regional referral hospital, to which patients are referred for antenatal problems such as miscarriage and complicated pregnancies and for management of difficult deliveries. The results of infant HIV PCR tests at birth were obtained from both sources as well as from a search of the computerised records of the NHLS, which is responsible for the testing of all public sector patients.

### Data analysis

Data were captured on a data sheet and transferred to a Microsoft Access database. Statistical analysis was performed using SPSS version 17.9.7 and MedCalc Statistical Software version 17.9.7. Missing data and outliers were verified for correct entry by checking the original source documents.

Categorical variables were compared with chi-square and Fisher’s exact tests as appropriate. Ordinal data were described by mean and standard deviation (s.d.) if normally distributed and by median, and by interquartile range (IQR) and range if not. Non-parametric data were compared with the Mann-Whitney U test.

## Ethical consideration

Permission to conduct the study was obtained from the University of KwaZulu-Natal Biomedical Research Ethics Committee (BE286/16).

## Results

Three hundred and eighty-six pregnant women presented for booking at the study site within the study period. One hundred and seventy three of these patients (44.8%) were HIV-positive and 94 (54.3%; 95% confidence interval [CI] 47% – 62%) were on PCART. Eighty-two participants (86% of those who potentially met inclusion criteria), selected on the basis of attendance on specific study days, were invited to participate and all enrolled. The mean age of the participants was 30.4 years (SD 6.1, range 18–43 years). The median gestational age at booking was 17 weeks (IQR 12–23, range 4–30). Eleven were first pregnancies (13.4%), and 72 were subsequent pregnancies (86.6%). Fifty-nine pregnancies (72%) were unplanned. The median duration of ART was 46 months (IQR 24–72, range 9–216). Seventy-two participants (87.8%) had disclosed their HIV status to their partner. Sixty-two participants (75.6%) were aware of their partner’s status. Of these, 42 partners were seropositive (67.7%) and 29 of the seropositive partners (69.0%) were themselves on ART.

**FIGURE 1:**
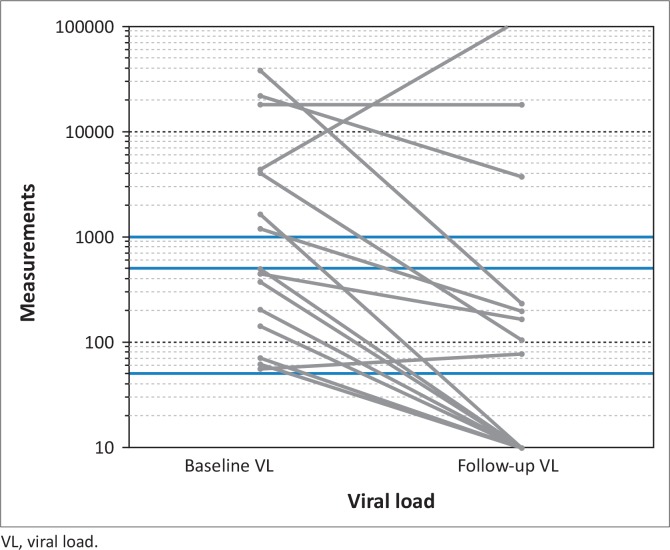
Evolution of viral load in 15 participants who were viraemic at the initial visit over the subsequent 4 weeks. The lower, middle and upper red lines represent viral load measurements of 50, 400 and 1000 copies/mL, respectively.

VL at enrolment and at the 4-week reassessment is shown in Table 1. Of 15 patients initially viraemic, seven showed evidence of viral suppression at the follow-up visit (Figure 1). Information on the outcome of pregnancy was available in 65 patients (79.3%) and is summarised in Table 2. Our participants reported three perinatal transmissions out of 141 live births in previous pregnancies, indicating a historical perinatal transmission rate of 2.1%.

**TABLE 1:** Viral load at baseline and at 4-week follow-up following intervention.

Viral load	CI	*n*	%
**At initial visit (*N* = 82)**			
Suppressed (< 50 copies/mL)	CI 71.3% – 89.1%	67	81.7
Viraemic (> 50 copies/mL)	CI 10.9% – 28.7%	15	18.3
< 400 copies/mL	-	6	7.3
• 400 copies/mL	-	9	11.0
**At 4-week follow-up (*N* = 22)**			
Suppressed (< 50 copies/mL) (*Transient viraemia*)	8.5% of total cohort	7	46.7
Viraemic (*Persistent viraemia*)	9.8% of total cohort	8	53.3
50–400 copies/mL	6.1% of total cohort	5	33.3
• 400 copies/mL	3.7% of total cohort	3	20.0

CI, 95% confidence interval (Wilson’s method corrected for continuity).

**TABLE 2:** Outcome of pregnancy in the study cohort.

Variable	All (*n* = 82)	No viraemia (*n* = 67)	Transient viraemia (*n* = 7)	Persistent viraemia (*n* = 8)
**Live**	62	49	6	7
Infant PCR positive	0	0	0	0
Infant PCR negative	62	49	6	7
**Spontaneous abortion**	4	4	0	0
**Stillbirth**	1	1	0	0
**Outcome unknown**	15	13	1	1

Of the 82 participants, 80 (97.6%) provided information on adherence using the VAS self-reported adherence test. The VAS score correlated significantly with the likelihood of viraemia. The median scores were 92.5 (IQR 30–100) and 100 (IQR 90–100) for the initial viraemia and suppressed group, respectively (*p* = 0.02) (Table 3). The VAS showed high specificity for initial viraemia on receiver operating characteristics (ROC) curve analysis (sensitivity 37.7% and specificity 98.5% at a cut-off value of 50, area under curve [AUC] 0.67). Four of the eight patients who demonstrated persistent viraemia (50%) reported submaximal follow-up VAS scores, while all six patients of the transient viraemia group (100%) scored themselves at 100. Pill counts proved unreliable, given that only 15 participants (18.3%) consistently brought their pills for counting. However, viraemic patients reported more missed doses in the preceding month than did those who were suppressed: the difference approached but did not reach statistical significance (*p* = 0.08).

**TABLE 3:** Correlation between self-reported adherence on initial visual analogue scale and viraemic status.

Adherence	Persistent viraemia		Transient viraemia		No viraemia	
	*n*	%	*n*	%	*n*	%
Full (100%)	3	37.5	3	50.0	46	69.7
Partial (< 100%)	5	62.5	3	50.0	20	30.3
Not reported	0	-	1	-	1	-

**FIGURE 2:**
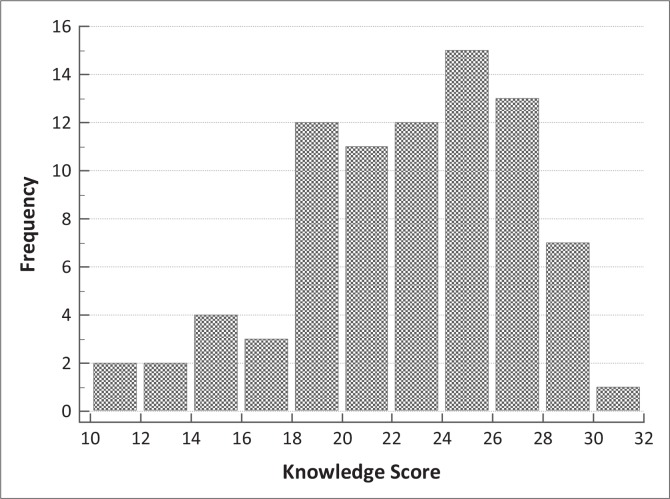
Distribution of knowledge scores among 82 participants. The maximum obtainable score is 32.

We assessed HIV knowledge by asking standardised questions on HIV disease, condom use and ART. We scored each of the 16 questions at 0, 1 or 2 points based on whether the participants showed no understanding, partial understanding or adequate understanding, respectively. We defined adequate understanding as recognition on the part of the participant of the importance of the knowledge item in self-management of HIV infection, as well as insight into the reasons for its importance, while no understanding implied either ignorance of the item or a lack of awareness of its significance. Partial understanding was intermediate, with some awareness of the knowledge item but no insight into its importance or relevance. We found knowledge to be generally good, with a median score of 23 of a possible 32 points (IQR 19–26, range 10–32) (Figure 2). The most poorly answered questions dealt with condom use, specifically the appropriate response to condoms breaking, negotiation of condom use when planning to conceive, responding to missed treatment doses, the relevance of VL monitoring and the development of antiretroviral drug resistance. We found a positive correlation of knowledge score with VAS score (*r* = 0.36, *p* = 0.0009). Patients with full VAS compliance scored higher than those with partial compliance (median 24 [IQR 21–26] vs. 20 [IQR 18–23], *p* = 0.002). We found no correlation between knowledge score in the viraemic group (median score 22 [IQR 19.3–24.7]) versus the fully suppressed group (median score 23 [IQR 21–25], *p* = 0.36).

We aggregated a number of questions in our questionnaire to produce four indicators of social deprivation. Thirty-seven (45.1%) involuntarily slept with more than three persons in a room, nine (11.0%) had involuntarily gone without food for two or more days in the preceding six months, 18 (22.0%) had not completed secondary school and 46 (59.1%) were currently unemployed. Only 13 (15.9%) of our sample had not experienced any of these challenges, and most participants had experienced multiple challenges (Table 4). However, none of these indices correlated significantly with either adherence or viraemia.

**TABLE 4:** Socio-economic indicators in 82 participants and their association with viraemia (transient or persistent) and full or partial adherence, as indicated by the self-assessed visual analogue scale.

Indicator	*N*	%	Association with viraemia	Association with adherence (VAS)
**Highest educational level**			*p* = 0.88	*p* = 0.86
Primary	18	21.9		
Secondary	35	42.7		
Tertiary	29	35.4		
**Employment status**			*p* = 0.74	*p* = 0.72
Currently employed or studying	36	43.9		
Current unemployed	46	56.1		
**Marital status**			*p* = 0.33	*p* = 0.81
Single	51	62.2		
Married, stable relationship or widowed	31	37.8		
**Consistent condom use**	12	15		
**Adverse socio-economic factors**			*p* = 0.79	*p* = 0.11
0 factors	13	15.9		
1 factor	21	25.6		
2 factors	26	31.7		
3 factors	16	9.5		
4 factors	6	7.3		

VAS, visual analogue scale.

The adverse socio-economic factors are overcrowding, food insecurity, failure to complete secondary schooling and unemployment.

We found no association between such parameters as gestational age, time on ART, previous PMTCT exposure, prior exposure to other ARV drugs, behavioural factors including condom use, alcohol use, disclosure and knowledge of partner status, and either adherence or viraemia (data not shown).

## Discussion

We found that 54% of the pregnant women seeking antenatal care at the study facility were receiving ART. Of women who booked on PCART, 18% presented with viraemia, and 47% of the viraemia was transient. Only 28% of pregnancies were planned. Viraemia at presentation was associated with non-adherence, as evidenced by low VAS scores for viraemic patients compared to those who were virally suppressed. Knowledge, socio-economic status and previous PMTCT exposure were not associated with viraemia.

The proportion of clinic attendees on ART is higher than those reported in earlier studies at 35% – 38%,^5,29^ in keeping with increasing ART coverage in the South African population. Most of our participants were unmarried, had had an unplanned pregnancy and booked later than recommended. The mean age was 30 years, which is consistent with data from South African ANC populations on ART. A significant number had not disclosed their HIV status to their partners. The unemployment rate at 56.1% is consistent with findings in other South African studies of both antenatal populations and general HIV-positive populations,^31,36,37^ and it may reflect relative disadvantage among the HIV-positive population.^38^

We found the prevalence of baseline viraemia to be 11.0% (CI 5.9% – 19.6%) at a threshold of 400 copies/mL and 18.3% (CI 11.4% – 28.0%) at 50 copies/mL. This is broadly consistent with experience in the general South African population of patients on ART and with the rates reported in South African studies of pregnant women on PCART, where prevalences of 22% – 23% have been reported at a threshold of 50 copies/mL^34,39^ and of 13% – 14% at a threshold of 400 copies/mL.^24,29^ Interestingly, Hoffmann et al.^40^ have shown that resuppression may be noted even where patients harbour drug-resistant virus.

Most of those who were initially viraemic showed a lower VL at the follow-up visit four weeks later (Table 1). Eighty per cent had a VL below 400 copies/mL and 47% a VL below 50 copies/mL. Our rate of viral resuppression is similar to the 44% and 56% at the 50 copies/mL and 400 copies/mL thresholds shown in other South African studies in pregnant patients.^25,29^ Retesting was preceded by adherence counselling without a change in treatment regimen, suggesting that initial viraemia may be at least in part a result of non-adherence.

Eight of our participants (9.8%) remained persistently viraemic, of whom five (63%) showed LLV below 400 copies/mL, and three (38% of the persistent group, 6% of the total cohort) had a VL exceeding 1000 copies/mL, suggesting virological failure. Cragg et al. reported a persistent viraemia rate of 3% at a threshold of 400 copies/mL,^29^ whereas Myer et al. reported that 22% of pregnant patients receiving ART remained persistently viraemic at a threshold of 50copies/mL.^26^ It is concerning that the rate of viral suppression reported for patients on ART in South Africa does not appear to have improved in the past decade. Three studies concluded in or before 2008 reported viraemia rates of 12% – 16% in patients on ART,^31,41,42^ not significantly different to our findings or to the other recent studies we quote. This is contrary to expectation. Current first-line ART regimens have better side-effect and toxicity profiles than previous regimens, which included stavudine and zidovudine, and the current pill burden of one pill per day in first-line ART is much lower than the burden of five or more pills of earlier regimens. Both these factors might be expected to improve adherence. Secondly, wider use of ART at community level could be expected to result in lower rates of reinfection in patients on ART as a result of lower levels of viraemia in HIV-positive sexual partners.^43^ Indeed, 69% of our participants’ partners who were reported to be HIV-seropositive partners were themselves on ART.

Recent reports have suggested a high prevalence of drug resistance in Southern African patients with ART failure on first-line regimens that contain tenofovir. This is frequently associated with the presence of thymidine analogue mutations and is frequently associated with resistance to other ARV drug classes as well.^44,45^ Numerous associations with viraemia in pregnant patients receiving ART have been reported in studies from Africa. These include ART duration, younger age, previous exposure to PMTCT, single status, non-disclosure of HIV status to their partner, illiteracy, a lower CD4+ count, concurrent TB treatment, ART commenced in third trimester of pregnancy and previous treatment default.^26,29,46^ Studies in higher-income countries, where the incidence of viraemia is lower, have found that vulnerable and disadvantaged populations are more likely to present with viraemia.^47^ Burch et al. found an association between viraemia and poor socio-economic circumstances, including unemployment, lack of university education, financial constraints and lack of stable housing in a British patient population.^48^ None of these associations were demonstrated in our study. The data from South Africa are conflicting. Azia et al. found economic constraints to be a barrier to adherence,^49^ while others^31,41^ failed to identify socio-economic challenges as barriers to good ART outcomes. That socio-economic factors may not be a major determinant of the success of ART in Africa is suggested by the low rates of viraemia in -ART-treated populations reported from Uganda of 11% at a threshold of 1000 copies/mL in a general population^46^ and 7% at a threshold of 400 copies/mL in pregnant patients,^50^ as well as a rate of 5% in an Ethiopian general population.^51^ Both these countries are of lower income than South Africa. McMahon et al. also found viral suppression rates in low-income countries to be comparable to high-income countries after 12 months of non-nucleoside reverse-transcriptase inhibitors (NNRTI)-based ART regimens.^52^ Thus, although our inability to show an association between adverse socio-economic circumstances and the probability of transient or persistent viraemia may have been methodological, there are indeed reasons to suspect that the lack of association is real.

Adherence was the only factor we found to be associated with viraemia in our cohort, suggesting that poor adherence is likely to be the major factor inducing transient viraemia. Non-adherence is known to be a strong predictor of viraemia, with significant odds ratios for non-adherence in four African studies ranging from 2.4 to 3.4.^5,41,46,51^

We found a significant association between initial viraemia and self-reported adherence as measured with a VAS, with a high specificity (98.5%) and low sensitivity (37.7%). Furthermore, of six participants who reported full adherence but were viraemic on initial presentation, three (50%) resuppressed following adherence counselling (Figure 2). It is possible that they too were non-adherent, particularly because self-reported measures are known to overestimate adherence.^53^ Of eight patients with initial viraemia who admitted partial adherence on VAS, only three (38%) resuppressed following counselling. This suggests either that they were persistently non-adherent, or that some other factor, including drug resistance, was operative. These samples will be tested for resistance. An important consideration is the reported evidence suggesting that viral suppression is more tolerant of partial adherence following a prolonged period of viral suppression.^41^ This implies that rigorous attention to adherence is particularly valuable early in the course of treatment. It is possible that some of the reportedly adherent participants with a detectable VL had failed to disclose their non-adherence, but in the absence of resistance testing this cannot be taken further.

We found that the VAS was a more reliable and practical measure of adherence than pill counts and 4- and 30-day recall. Chaiyachati et al. undertook a study comparing the predictive accuracy of five measures of non-adherence in predicting treatment failure in a community in rural KwaZulu-Natal.^54^ They found the VAS to be highly specific (98% – 100%, dependent on the threshold used), although with low sensitivity (0% – 7%) in predicting adherence. Indeed, they found that no method had both acceptable sensitivity and acceptable specificity, and the accuracy of the VAS was not inferior to any other method in terms of either sensitivity or specificity.

Although the level of knowledge shown by our participants is reassuring, it is of concern that some participants were not aware about post-exposure prophylaxis and emergency contraception in the event of condoms breaking, nor about how to safely negotiate condom use when planning conception. We found our cohort to lack sufficient knowledge about the danger of non-adherence. The knowledge shown by our participants is consistent with that shown in other KwaZulu-Natal studies.^55,56^ Haffejee et al., in their study of women in a low-income community, found that although women had general knowledge about HIV they lacked knowledge of specific prevention behaviour.^56^ Mindry et al. found that ART clinic patients had insufficient knowledge on safe conception methods.^55^

Ninety-three per cent of pregnancies in our study whose outcomes were known resulted in live births (Table 2). We were unable to determine the outcomes of 18% of pregnancies in our cohort. These were patients who delivered at institutions other than the study ANC or its referral hospital, whose infant PCR could not be identified through laboratory records on a search by mother’s identifying data and who were not contactable by telephone.

None of the infant birth PCR results showed inutero HIV transmission (Table 2). This study did not measure viraemia at the time of delivery. Confidence intervals are wide given our small sample size, and thus caution is needed in interpreting our finding of zero MTCT. However, we believe it probable that very few if any of our participants would have been viraemic at the time of labour, given that the patients with transient viraemia had resuppressed, while those with persistent viraemia were referred for further intervention, including a change in regimen. Even the historical perinatal transmission rate of 2.3% reported by our participants for their previous pregnancies is much lower than the rate of MTCT of approximately 15% – 45% recorded in the pre-ART era,^20^ in keeping with much improved outcomes with modern approaches.

We believe our study is valuable in that it prospectively followed a group of patients on PCART, as opposed to those commencing ART in pregnancy. It also sought to correlate viraemia with possible causal factors, including adherence and socio-economic disadvantage. However, it is subject to a number of limitations, particularly in that it was a cross-sectional study and that only the viraemic group had follow-up VL taken to determine persistence or transience of viraemia. Serial VL measurement in all patients at regular intervals until delivery would be necessary to understand the natural history of viraemia in pregnancy fully. The factors driving loss of viral suppression were not rigorously identified. Infant PCR results were incomplete, a consequence of a fragmented healthcare system with poor continuity of care.

With an increasing number of perinatally infected girls born in the era before comprehensive ART coverage now reaching childbearing age, and increasing national ART coverage expected with the Universal Test and Treat initiative,^57^ the number of women entering ANC on PCART will rise. Our findings suggest that 80% of women on PCART accessing antenatal care in a South African semirural setting are virologically suppressed. Though below the aspirational target of 90% suppression rate by 2020,^16^ it is encouraging, as is the zero MTCT rate we noted.

There is a need for further study, including qualitative studies to delineate both the causal factors driving viraemia in pregnancy and its natural history, particularly in terms of the development of maternal virological failure and the rate of vertical transmission. Viraemia rates are still too high, and the factors responsible for this need to be identified and their prevention built into PMTCT programmes, including attention to upstream factors such as education and the prevention of unplanned pregnancies.^58^

We identified two areas of particular concern. Firstly, 72% of pregnancies were unplanned in a population of patients on ART, thus depriving them and their partners of the opportunity for protective measures such as timed intercourse, planned interruption of regular condom use and pre-exposure prophylaxis for the non-infected partner. Even in the remaining 28%, we found no evidence of planning of conception in collaboration with healthcare service providers. Secondly, we were unable to determine birth outcomes for 18% of our pregnancies despite our best efforts, which speaks to the fragmentation of our healthcare system even within the PMTCT cascade, as well as serious deficits in communication and sharing of critical information between providers.

Finally, we have shown that self-reported non-adherence using the VAS has value. The VAS is not commonly employed for the assessment of adherence in our environment. However, it is a quick and simple method and should be developed further as part of routine patient assessment.

## Conclusion

One-fifth of HIV-positive pregnant women on PCART are viraemic at antenatal booking. ART programmes miss the opportunity to either optimise viral suppression in this group of women before conception occurs or to offer contraception services. Non-adherence is a very important factor in viraemia, and a concerted effort by both healthcare workers and patients is necessary to achieve optimum adherence to ART. The rate of unplanned pregnancies is high; therefore, more action is required to meet the contraception needs of HIV-positive women as pregnancy prevention is an effective means of PMTCT. More effective integration of such services as family planning, ARV treatment, reproductive health, maternal and neonatal services, as recommended in national guidelines, is necessary.^59^
